# Modulating Smart Mechanoluminescent Phosphors for Multistimuli Responsive Optical Wood

**DOI:** 10.1002/advs.202305066

**Published:** 2023-11-08

**Authors:** Jiangcheng Luo, Biyun Ren, Xianhui Zhang, Mingju Zhu, Tianlong Liang, Zefeng Huang, Yuantian Zheng, Xu Li, Jianwei Li, Zitong Zheng, Bing Chen, Yu Fu, Dong Tu, Yu Wang, Yanmin Jia, Dengfeng Peng

**Affiliations:** ^1^ Key Laboratory of Optoelectronic Devices and Systems of Ministry of Education and Guangdong Province College of Physics and Optoelectronic Engineering Shenzhen University Shenzhen 518060 China; ^2^ Faculty of Materials Science and Chemistry China University of Geosciences Wuhan 430074 China; ^3^ SZU‐NUS Collaborative Innovation Center for Optoelectronic Science & Technology International Collaborative Laboratory of 2D Materials for Optoelectronics Science and Technology of Ministry of Education Institute of Microscale Optoelectronics Shenzhen University Shenzhen 518060 China; ^4^ School of Physics and Information Technology Shaanxi Normal University Xi'an 710062 China

**Keywords:** long afterglow phosphor, mechanoluminescence, optical wood, smart materials, Sr_2_MgSi_2_O_7_

## Abstract

Mechanoluminescence is a smart light‐emitting phenomenon in which applied mechanical energy is directly converted into photon emissions. In particular, mechanoluminescent materials have shown considerable potential for applications in the fields of energy and sensing. This study thoroughly investigates the mechanoluminescence and long afterglow properties of singly doped and codoped Sr_2_MgSi_2_O_7_(SMSO) with varying concentrations of Eu^2+^ and Dy^3+^ ions. Subsequently, a comprehensive analysis of its multimode luminescence properties, including photoluminescence, mechanoluminescence, long afterglow, and X‐ray‐induced luminescence, is conducted. In addition, the density of states mapping is acquired through first‐principles calculations, confirming that the enhanced mechanoluminescence properties of SMSO primarily stem from the deep trap introduced by Dy^3+^. In contrast to traditional mixing with Polydimethylsiloxane, in this study, the powders are incorporated into optically transparent wood to produce a multiresponse with mechanoluminescence, long afterglow, and X‐ray‐excited luminescence. This structure is achieved by pretreating natural wood, eliminating lignin, and subsequently modifying the wood to overall modification using various smart phosphors and epoxy resin composites. After natural drying, a multifunctional composite wood structure with diverse luminescence properties is obtained. Owing to its environmental friendliness, sustainability, self‐power, and cost‐effectiveness, this smart mechanoluminescence wood is anticipated to find extensive applications in construction materials and energy‐efficient displays.

## Graphical abstract

Mechanoluminescent phosphors are considered smart materials for a myriad of applications. In this work, the multistimuli responsive Sr_2_MgSi_2_O_7_ phosphor are experimentally modulated and theoretically studied. After that, the selected smartphosphors are successfully integrated into transparent woods, which offer great promise for various applications in the fields of self‐diagnosis buildings and energy‐efficient lighting and advanced self‐powered displays.

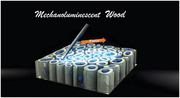

## 1 Introduction

Mechanoluminescence (ML) is the direct conversion of mechanical energy into light emission.^[^ ^1^, ^2^, ^3^ ^]^ As a magical light source, ML offers a novel approach to light emission without requiring extra electrical energy input. ML objects are considered smart materials for valuable applications in various fields, including structural health diagnosis of large engineering steel frames or civil structures.^[^ ^4^, ^5^, ^6^ ^]^ Natural mechanical energy includes wind‐or water‐driven light‐emitting,^[^ ^7^, ^8^, ^9^ ^]^ stretchable and flexible displays,^[^ ^10^, ^11^, ^12^, ^13^ ^]^ artificial skin,^[^ ^14^, ^15^ ^]^ athletic analytics in sports science,^[^ ^6^, ^16^, ^17^ ^]^ human–computer/machine interaction,^[^ ^18^, ^19^, ^20^ ^]^ ultrasound‐activated luminescence for noninvasive optogenetics and sound seeing,^[^ ^21^, ^22^ ^]^ battery‐free display and energy‐saving lighting for the traffic^[^ ^6^, ^23^ ^]^ and mechano‐optic/piezo‐photonic/tribo‐photonic anti‐counterfeiting,^[^ ^24^, ^25^, ^26^, ^27^, ^28^ ^]^ and several new advanced applications under development. Unlike the current mechanisms in most organic luminescent materials, which rely on chemical bond breaking for luminescence, mechanoluminescence in inorganic materials offers superior repeatability. This is achieved by utilizing piezoelectric and friction effects, allowing for a stable and highly luminous output even under repeated stress conditions.^[^ ^11^ ^]^ Some ML materials can be viewed as a new type of smart phosphor with multiple response characteristics, memory characteristics,^[^ ^29^, ^30^ ^]^ biomimetic properties,^[^ ^31^ ^]^ super‐sensitive stress response properties,^[^ ^32^, ^33^ ^]^ and reproducible/self‐recovery properties,^[^ ^34^, ^35^ ^]^ which will play an essential role in future applications in multiple areas.

Sr_2_MgSi_2_O_7_ (SMSO) is a prominent and exemplary inorganic silicate phosphor that has been extensively investigated in previous studies.^[^ ^36^, ^37^, ^38^, ^39^, ^40^, ^41^ ^]^ This material exhibits several remarkable luminescent properties, including ML, long afterglow (AG), and photoluminescence (PL). SMSO has been used as a commercial blue long afterglow material and has started to be applied in several fields. For example, in terms of mechanoluminescence, Hong et al. recently realized ultrasonically activated vibrating ML using the biomineral‐inspired suppressed dissolution method,^[^ ^40^, ^41^ ^]^ and Zhang et al. utilized the long afterglow luminescence properties of SMSO as an all‐weather catalyst for selectively reducing CO_2_ to CO.^[^ ^38^ ^]^ SMSO, as a smart phosphor that has received widespread attention, lacks systematic research on its material composition for ML and AG, such as the optimal experimental conditions for luminescence, doping ratio, and the repeatability and stability of luminescent properties. In‐depth research and exploration of these aspects would be beneficial for expanding related applications, such as stress sensing for health structure diagnosis and environmental auxiliary lighting,^[^ ^42^ ^]^ as well as for expanding composite and hybrid bonding with other functional materials.^[^ ^6^ ^]^

The utilization and development of traditional natural materials have become increasingly popular in recent years.^[^ ^8^ ^]^ Wood is one of the most abundant biological resources available in nature and offers numerous advantages, such as high economic efficiency, environmental friendliness, a low carbon footprint, and excellent mechanical stability and durability. Its layered and porous structure and lignocellulosic composition facilitate efficient material transport and provide a substantial load‐bearing capacity. This unique material structure opens new avenues for the development of functional materials, and enhancing the properties of wood has emerged as a significant research area.^[^ ^43^, ^44^ ^]^ Chemical modifications are a well‐established approach. The chemical modification involves the removal of lignin from natural wood using NaOH, Na_2_SO_3_, or H_2_O_2_. This strategy leads to the production of transparent“optical wood”.^[^ ^45^, ^46^, ^47^ ^]^ By eliminating the light‐absorbing components of wood and lignin and infusing organic matter to match the refractive index of cellulose, wood can achieve transparency while simultaneously enhancing its mechanical properties.^[^ ^48^, ^49^, ^50^ ^]^ This results in wood with excellent physical properties, making it suitable for various applications, including building materials and furniture, and for further matrices used for functional filling, such as photoluminescent and electroluminescent wood,^[^ ^51^, ^52^, ^53^ ^]^ making it possible to design mechanoluminescent wood that emits photons under mechanical stimuli. Inspired by these ideas, we successfully combined SMSO and epoxy resin with wood strips, resulting in wood that possesses mechanically induced ML and AG properties. This innovative wood can serve as a novel light source, intelligent display, and anti‐counterfeiting medium for writing.

Herein, we present a comprehensive study of the ML and AG performance of Eu^2+^ and Dy^3+^ under different doping concentrations to determine the optimal concentration for the best performance. SMSO was synthesized using a high‐temperature solid‐phase method by varying the doping concentrations of Eu^2+^ and Dy^3+^ to modify and optimize the ML and AG properties. The multiluminescence properties of the synthesized SMSO samples were characterized, including long afterglow, lifetime, quantum yield, thermal luminescence, and ML. The density of states before and after SMSO doping was determined through theoretical calculations, allowing the evaluation of trap depths within the material. These calculations provide valuable insights into the electronic structure and properties of the doped SMSO. By incorporating the prepared SMSO powders, we successfully obtained mechanoluminescent wood that demonstrated both intrinsic wood properties and mechanical‐to‐optical energy conversion properties.

## 2 Results and Discussion

**Figure 1:**
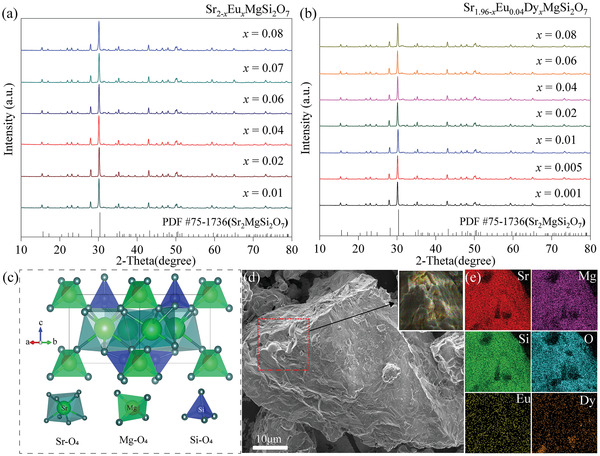
Crystal structure and morphology characterization of the SMSO phosphors. a,b) X‐ray diffraction (XRD) patterns of Sr_2‐x_Eu_x_MgSi_2_O_7_ (*x* = 0.01, 0.02, 0.04, 0.06, 0.07, and 0.08) and Sr_1.96‐x_Eu_0.04_Dy_x_MgSi_2_O_7_ (*x* = 0.0001, 0.001, 0.005, 0.01, 0.02, 0.04, 0.06, and 0.08) samples, respectively; c) Schematic illustration of the crystal structure of SMSO; d)Typical SEM image of the SMSO:2% mol Eu^2+^, 2% mol Dy^3+^ sample; e) Energy‐dispersive X‐ray spectroscopy (EDS) images of the corresponding enlarged area shown in (d).

In a representative experiment, we synthesized single‐doped and co‐doped lanthanide SMSO phosphors using a solid‐state method to study the impact of doping on structural characteristics. **Figure** **1**a,b depict the X‐ray diffraction (XRD) patterns of the Eu‐doped SMSO and Eu‐ and Dy‐codoped SMSO powders with different doping concentrations. These patterns exhibited characteristic peaks indexed in accordance with the SMSO phase (PDF#75‐1736), indicating pure‐phase products with high crystallinity. The effects of introducing Eu and Dy dopants on the crystallinity of the substrate were investigated. However, the XRD patterns remained consistent across different doping concentrations, suggesting that the presence of these dopants had minimal influence on the crystalline structure. Even at relatively high doping concentrations, such as 2% Eu^2+^/2% Dy^3+^ (Eu^3+^ ions were reduced to Eu^2+^ by an H_2_/N_2_ atmosphere), the XRD patterns consistently displayed diffraction peaks that aligned with the standard card, indicating the high crystallinity of the SMSO. To better understand the crystal structure parameters, the Rietveld XRD refinement of two representative samples was performed, as shown in Figure S1 (Supporting Information). The profile factors obtained for SMSO:2% Eu^2+^ were *R*_p_ = 7.9%, *R*_wp_ = 10.77%, and χ2 = 2.24. Similarly, for SMSO:2% Eu^2+^/2% Dy^3+^, the profile factors were *R*_p_ = 9.8%, *R*_wp_ = 12.59%, and χ2 = 1.7, indicating a satisfactory linear fit. The similarity in ionic radius and electronic structure between Eu^2+^ (1.25 Å) and Dy^3+^ (1.02 Å) dopants, as well as Sr^2+^(1.26 Å) ions in SMSO, allows for preferential substitution of the dopants at the Sr sites, this substitution results in changes in lattice parameters owing to the differing ionic radii and occupancy of the dopants. The lattice parameters of the undoped SMSO phosphors, 2% mol Eu^2+^‐doped SMSO, and 2% mol Dy^3+^‐codoped SMSO are provided in Table S1 (Supporting Information).

SMSO is a typical yellow feldspar with a space group of P421m, as shown in Figure 1c. The structure reveals a distinctive layered arrangement, where one layer is marked by the presence of anionic polyhedra formed by interconnected Mg─O_4_ and Si─O_4_ tetrahedra through diagonal connections. The adjacent layer comprises cationic Sr–O_8_ units, which are interconnected in a coangular fashion. The molecular structure of SMSO is interconnected through reactive oxygen species, which foster strong bonding interactions. In addition, the overall molecular structure exhibits tetragonal symmetry, further highlighting the structural organization and characteristics of SMSO. Figure 1d shows the scanning electron microscope (SEM) image of SMSO phosphors subjected to secondary calcination, including the first calcination for 2 h at 900 °C and secondary calcination process for 4 h at 1400 °C under a reducing atmosphere. Furthermore, additional SEM images are shown in Figure S2 (Supporting Information), illustrating the samples after primary calcination for 2 h at 1100 °C and samples treated after secondary calcination for 2 and 6 h at 1100 and 1400 °C, respectively. Incorporating these images provided a comprehensive view of the morphological changes in the SMSO induced by varying the calcination conditions. The samples initially appeared spherical after the primary calcination and subsequently agglomerated to form large flakes. Energy‐dispersive X‐ray spectroscopy (EDS) performed on the selected area in Figure 1e demonstrates a homogeneous distribution of Sr, Mg, Si, O, Eu, and Dy within the analyzed region. This observation provides compelling evidence that the desired samples were successfully synthesized, confirming the efficient incorporation of the dopant ions into the SMSO crystal structure. The uniform elemental distribution further confirmed the formation of a well‐integrated and chemically homogeneous material, thereby reinforcing the reliability and quality of the synthesis process.

**Figure 2:**
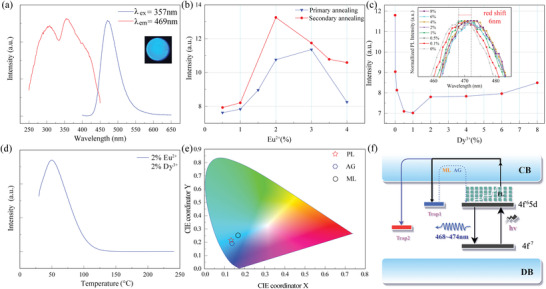
PL studies on the SMSO. a) Photoexcitation and emission spectra of the SMSO:2% Eu^2+^/2% Dy^3+^ phosphors; b) Relationship between the Eu^2+^ doping concentrations and the integrated intensity of the 400–600 nm emission spectrum of the samples after primary and secondary calcination; c) Relationship between Dy^3+^ concentrations and integrated emission intensity, the inset graph shows the change of the emission peak; d) Thermoluminescence curve of the SMSO; e) CIE diagram of SMSO spectra; f) Luminescent mechanism of PL, AG, and ML.

Subsequently, the PL properties of the SMSO samples were investigated, and the results are shown in **Figure** **2**. The excitation wavelength of SMSO was determined to be within the range of 250–450 nm, which including the blue region, thus demonstrating its capability to be efficiently excited by widely available light sources such as sunlight, Ultraviolet (UV)/blue or white LEDs, cell phone panels, and flashlights. The incorporation of Eu^2+^ dopants in SMSO yielded a distinct instinct emission band spanning 400–600 nm, exhibiting a prominent peak at ≈470 nm, indicating the generation of high‐quality blue light with significant energy content (Figure 2a). As Figure 2b illustrates, the influence of the Eu^2+^ doping concentration on the PL intensity was systematically investigated by integrating the emission spectrum ranging from 400–600 nm and measuring the corresponding intensity at various doping concentrations.

Notably, distinct optimal doping concentrations for photoluminescence were observed for the samples subjected to primary and secondary calcination processes. Specifically, the optimal doping concentration for samples after primary calcination was determined to be 3% mol, whereas, for samples after secondary calcination, the optimal concentration was 2% mol, indicating that multiple sintering cycles can influence the optimum concentration of Eu. Surpassing the optimal doping concentration reduced the integrated intensity, emphasizing the importance of maintaining an appropriate doping level. Moreover, the integrated intensity after secondary calcination exceeded that after primary calcination. This divergence can be attributed to the insufficient reaction time during the primary calcination process, wherein the reaction between the carbonate and silicon oxide raw materials requires an additional duration for adequate progression. The inclusion of a secondary grinding step enhanced the contact between the raw materials, thereby facilitating a more comprehensive incorporation of dopant elements into the crystal structure. Consequently, this variance in the processing stages contributed to the emergence of diverse optimal doping concentrations, underscoring the crucial role played by the reaction time and material interactions in dictating the luminescent characteristics and optimal doping requirements. The effect of Dy^3+^ doping on the photoluminescence properties was investigated, and the results are shown in Figure 2c and Figure S3 (Supporting Information). Notably, incorporating Dy^3+^ initially decreased the emission intensity, followed by an increase, culminating in a stable position. This behavior can be attributed to introducing deeper trap levels facilitated by Dy^3+^ doping, leading to the capture of a greater number of electrons within the Dy^3+^ energy level, thereby impeding their immediate escape. The impact of the Dy^3+^ doping concentration on the emission peak was investigated. The results revealed a noticeable trend wherein the emission peak shifted slightly to ≈3–5 nm toward longer wavelengths with increasing doping concentration. This shift can be attributed to substituting Dy^3+^ and Eu^2+^ ions at the Sr^2+^ sites. Notably, Dy^3+^ possesses a distinct ionic radius compared to Sr^2+^, and its incorporation into the lattice introduces structural perturbations. As the concentration of the Dy^3+^ dopant increased, the c‐axis length decreased. This alteration in the lattice parameters facilitates a more advantageous and quantitatively favorable arrangement of the d orbitals. Consequently, the emission peak was redshifted, indicating a lengthened emission wavelength.

The quantum yield (QY) is an important indicator of phosphor luminescence performance. The QY of the samples was measured to assess their efficiency in converting the absorbed photons into emitted photons. Remarkably, the sample synthesized through a single‐step calcination process exhibited the highest QY of 70.77%. However, the adoption of a two‐step doping strategy decreased the QY, and the subsequent introduction of Dy^3+^ further diminished the QY to 57.27% (Table S2, Supporting Information). These findings suggest that the presence of Dy^3+^ significantly influenced the luminescence behavior and QY of the synthesized samples. The observed decrease in the quantum yield can be attributed to the combined effects of complex energy transfer processes and introduction of deeper energy levels associated with Dy^3+^ doping. This insight is critical for tailoring the photoluminescent properties and optimizing the quantum efficiency of material for potential applications in various optoelectronic devices.

Figure 2d illustrates the thermoluminescence (TL) curve obtained for the samples of SMSO doped with 2% Eu^2+^ and 2% Dy^3+^. The TL curve displays a distinct peak at ≈46 °C. Utilizing the Hoogenstraaten method, we employed an estimation technique to determine the trap depth, which yielded a value of ≈0.48 eV.

The emission peaks at 470 nm were obtained at excitation wavelengths of 250–450 nm. However, the emission intensities differed, with the strongest emission peak observed under 357 nm excitation. This observation suggests the presence of a singular luminescence center that aligns with the structural configuration of a solitary Sr‐ion site within the host matrix. This distinction arises from the selective replacement of Sr atoms with Eu^2+^, wherein variations in the ionic radii influence the occupancy behavior. Consequently, discrepancies arose in the splitting of the 5d energy levels of the Eu^2+^ ions, leading to modifications in the excitation spectrum. Moreover, the energy transfer from the Eu^2+^ ion with higher emission energy to the Eu^2+^ ion exhibiting lower absorption energy further contributes to alterations in the excitation spectrum. Interestingly, we found that the PL of SMSO has polarization characteristics, as shown in Figure S4 (Supporting Information). By adjusting the line polarizer, the intensities of the two peaks were changed. This may be due to the hand‐lettering phenomenon. The polarization phenomena were not observed in ML or AG.

As shown in Figure 2e, we measured ML, AG, and PL in SMSO and obtained luminescence curves that exhibited similar characteristics. The CIE coordinates indicate that the PL emissions have coordinates (0.1366, 0.1956) and AG emissions have coordinates (0.1333, 0.2144), whereas the ML emissions have slightly shifted coordinates (0.1668, 0.2519). The luminescence behavior of SMSO with respect to ML, PL, and AG was elucidated based on previous research findings, and the corresponding mechanism is illustrated in Figure 2f. Upon UV excitation, a photon undergoes a transition from the 4f ^7^ state to the 4f ^6^5d state. Subsequently, partial radioactive decay occurs, causing the system to revert from the 4f ^6^5d state to the 4f ^7^ state. Consequently, the light emitted at 470 nm corresponded to the PL mechanism exhibited by the SMSO. Some photons traverse the conduction band and are trapped by defects. In this context, the presence of Dy^3+^ imparts deeper traps capable of capturing a larger number of charges. At room temperature, the trapped electrons in shallow and deep traps are activated, producing AG emissions. Furthermore, when an external force is applied to the SMSO, a pyrite‐like crystal is generated owing to its remarkable piezoelectric properties. This effect facilitates the release of electrons, particularly from deeper traps. Subsequently, these released electrons migrate to the luminescence centers, giving rise to the ML phenomenon observed in SMSO.

**Figure 3:**
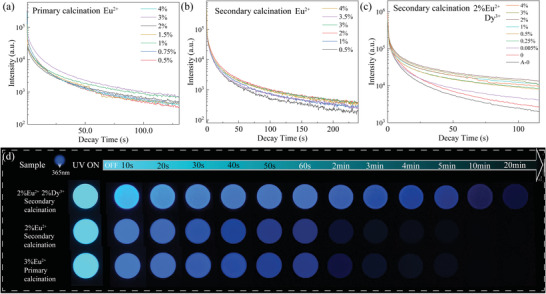
Investigation of the afterglow properties of SMSO. a) Afterglow curves of different concentrations of Eu^2+^‐doped SMSO under 357 nm excitation in the case of one‐step calcination; b) Afterglow curves of different concentrations of Eu^2+^ after 357 nm excitation in the case of two‐step calcination; c) Afterglow curves of different concentrations of Dy^3+^ under 357‐nm excitation in the case of two‐step calcination; d) Afterglow images were captured for various concentrations of doped samples with Polydimethylsiloxane (PDMS) complexes. These images were acquired at different intervals after a 2‐min excitation at 365 nm.

SMSO are commonly used in energy storage and discharge applications.^[^ ^37^ ^]^ Notably, the SMSO exhibited residual glow emission with a characteristic wavelength of 470 nm. Upon exposure to UV irradiation, the electrons residing in the ground state (4f) of the Eu ions absorb incident photons, which induce a transition to the conduction band (5d) and subsequently lead to the conversion of Eu^2+^ to Eu^3+^. These excited electrons are entrapped at the oxygen hole sites along their trajectory within the conduction band. As shown in **Figure** **3**a,b, the optimal doping concentration was 3% mol Eu^2+^ under one‐step calcination and 2 mol Eu^2+^ under the two‐step method, based on which we codoped into the study of the effect of Dy^3+^ Incorporation of Dy^3+^ doping species introduces deeper trapping states, thereby impeding the prompt escape of the captured electrons. Incorporating Dy^3+^ dopants into the SMSO notably influenced the afterglow characteristics, as shown in Figure 3c. The afterglow intensity demonstrated pronounced enhancement with increasing Dy^3+^ concentration, reaching a maximum of 2% mol.

Figure 3d shows the selected samples of the highest intensity from each series: 3% mol Eu^2+^ obtained through one‐step calcination, 2% mol Eu^2+^ obtained through two‐step calcination, and a composite product comprising 2% mol Eu^2+^ and 4% mol Dy^3+^ mixed with PDMS. The phenomenon shown in the figure was achieved by subjecting the samples to irradiation under a 365 nm lamp for 1 min. Notably, the sample lacking Dy^3+^ doping experiences a substantial decline in brightness within 2 min, whereas the sample with the optimal concentration of Dy^3+^ doping maintains a visible afterglow for more than 10 min. This observation highlights the influential role of Dy^3+^ doping on the afterglow persistence of the samples. The presence of Dy^3+^ not only enhances the initial afterglow intensity but also prolongs its duration, leading to superior performance compared to the nondoped sample.

Compared to aluminate compounds renowned for their strong afterglow effects, silicate afterglow materials, particularly SMSO, exhibit exceptional afterglow properties while maintaining remarkable chemical stability. In Figure S5 (Supporting Information), the specimens underwent immersion in water for 7 days at three distinct temperatures: 0 °C, 80 °C, and room temperature. Comparative analysis indicates that the residual glow effect did not diminish, thereby substantiating the outstanding environmental stability of the SMSO. Conventional photovoltaic energy storage materials,^[^ ^23^ ^]^ such as strontium aluminate, progressively undergo hydrolysis when exposed to water, leading to a gradual decline in afterglow performance. Addressing this issue typically entails the application of moisture‐resistant coatings, such as Al_2_O_3_ or TiO_2_, which significantly increases production costs. However, SMSO demonstrates laudable stability and persists in exhibiting afterglow characteristics even when subjected to routine scenarios encountered in daily life, including water immersion and exposure to varying temperatures. The robust environmental resilience displayed by the SMSO suggests its suitability for diverse practical applications without experiencing degradation across different environmental conditions.

**Figure 4:**
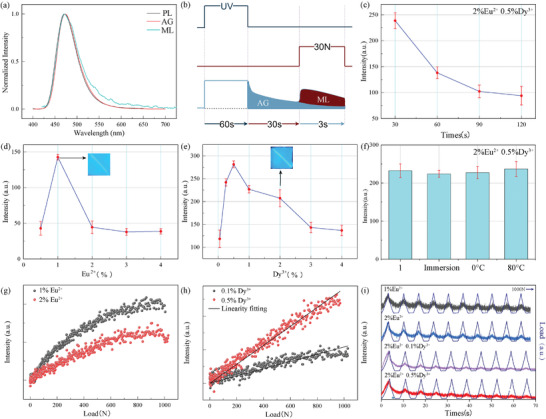
ML properties of SMSO. a) Normalized AG, ML, and PL spectra of SMSO; b) Schematic of the ML measurement method; c) Natural decay after sufficient irradiation, decay time versus ML integrated intensity; d) Relationship between Eu^2+^ doping concentration and ML integrated intensity at 30 N force; e) Relationship between Dy^3+^ doping concentration and ML integrated intensity at 30 N force; f) ML intensity of SMSO at four different conditions: room temperature, water immersion, 0 °C, and 80 °C; g) ML intensity of two‐step calcination of SMSO 1% mol Eu^2+^ and 2% mol Eu^2+^ at high pressure: 0–1000 N force; h) ML intensity of two‐step calcination of SMSO‐doped 2% Eu^2+^/0.1% Dy^3+^ and 2% Eu^2+^/0.5% Dy^3+^ (mol ratio) at high pressure: 0–1000 N force; i) ML intensity of two‐step calcination 1% Eu^2+^, 2% Eu^2+^, and 2% Eu^2+^/0.1% Dy^3+^, 2% Eu^2+^/0.5% Dy^3+^ (mol ratio) under high‐pressure cycles of 0–1000 N.

**Figure** **4**a shows the ML spectrum obtained from SMSO during the investigation of the ML of SMSO. The observed spectrum shows an emission profile similar to the PL and AG spectra. This similarity strongly suggests that ML originates from the transitional process between the 4f ^7^ and 4f ^6^5d energy levels of the Eu^2+^ ion emission center. As shown in Figure 4b, ML measurement was performed using a precise procedure. The SMSO sample was irradiated with a 365 nm UV lamp for 1 min. Subsequently, the sample was allowed to undergo natural decay for 30 s, during which the AG intensity was recorded from the 30th to the 33rd second. Subsequently, a controlled force of 30 N was applied to induce a sliding motion on the sample between the mentioned time intervals. The actual stress intensity can be accurately determined by subtracting the recorded AG intensity from the combined intensity of the mechanical emitter and afterglow.

To obtain reliable and statistically significant data, four measurements were performed on the same sample to ensure the accuracy and reproducibility of the results. This experimental approach was selected to enhance the signal‐to‐noise ratio and minimize potential experimental errors arising from variations in the decay times. As shown in Figure 4c, an investigation was conducted to explore the correlation between the different decay times and the intensity of the mechanical luminophores following 1‐min irradiation. The results demonstrated an initial decrease in the ML intensity, followed by stabilization as the decay time increased. From these findings, it can be inferred that the optimal measurement time is 30 s. At this point, the ML luminous intensity was at its peak, whereas the interference from the background noise and potential experimental uncertainties causing errors were minimized. Consequently, the signal‐to‐noise ratio was maximized, enhancing overall experimental accuracy.

Figure 4d shows the correlation between the ML intensity of SMSO and Eu^2+^ doping concentration. The findings reveal that the ML intensity reaches its peak at 1 mol.% Eu^2+^ doping concentration. Moreover, the ML intensity was investigated at various Dy^3+^ doping concentrations while maintaining a 2 mol.% Eu^2+^ base. As shown in Figure 4e, the results demonstrated that the optimal ML intensity was achieved at a 0.5 mol.% Dy^3+^ concentration. Notably, increasing the doping concentration led to a significant enhancement in the ML intensity. Adding an appropriate amount of Dy^3+^ doping augmented the overall brightness and ML luminescence intensity of SMSO owing to the introduced trapping effect. Deeper traps can encapsulate more electrons, resulting in more robust light emission when subjected to external forces and the generation of a piezoelectric field. The conspicuous blue light produced during sliding beneath a glass rod is discernible to the naked eye, underscoring the exceptional potential of SMSO in diverse everyday applications such as sensing and anti‐counterfeiting. To assess the influence of environmental factors on the ML intensity, the SMSO sample doped with 2 mol.% Eu^2+^ and 0.5% mol Dy^3+^ was partitioned into four distinct portions. In Figure 4f, these portions underwent exposure to various environmental conditions for seven days, including room temperature, water immersion, 0 °C, and 80 °C. Figures 4g,h illustrate the ML response of the SMSO particles when subjected to a 1000 N load during the initial force cycle. The ML intensity exhibited a gradual and proportional increase with increasing applied stress. Dy^3+^ doping of the sample enhanced the linearity effect compared to that of the undoped sample. The demonstrated linearity between stress and the visually prominent ML response under everyday pressure conditions highlights the excellent sensing potential of SMSO. After the designated period, the samples were meticulously evaluated, and it was observed that their ML intensities remained consistently stable across all tested environmental conditions. Figure 4i was utilized to evaluate the repeatability of the samples subjected to significant stress levels. A total of 10 cycles were performed, involving varying pressures ranging from 0 to 1000 N and then returning to 0 N. The ML‐stable luminescence of the samples containing 1% Eu^2+^, 2% Eu^2+^, 2% Eu^2+^/0.1% Dy^3+^, and 2% Eu^2+^/0.5% Dy^3+^ (mol) was measured and recorded. However, the stress intensity gradually declined over the cycles owing to the release of electrons from the trapped states. Notably, during the experiment, the ML intensity of the 0.5% mol Dy^3+^‐doped sample decreased to ≈31% of its initial intensity by the tenth cycle. By contrast, the sample doped solely with 2% mol Eu^2+^ experienced y smaller decline to only 17% of the original intensity. When considering the undoped Dy^3+^ sample, a notable reduction in the ML intensity was observed by the 10th cycle, whereas the Dy^3+^‐doped sample exhibited a moderate decrease, which was attributed to the heightened capture of electrons.

**Figure 5:**
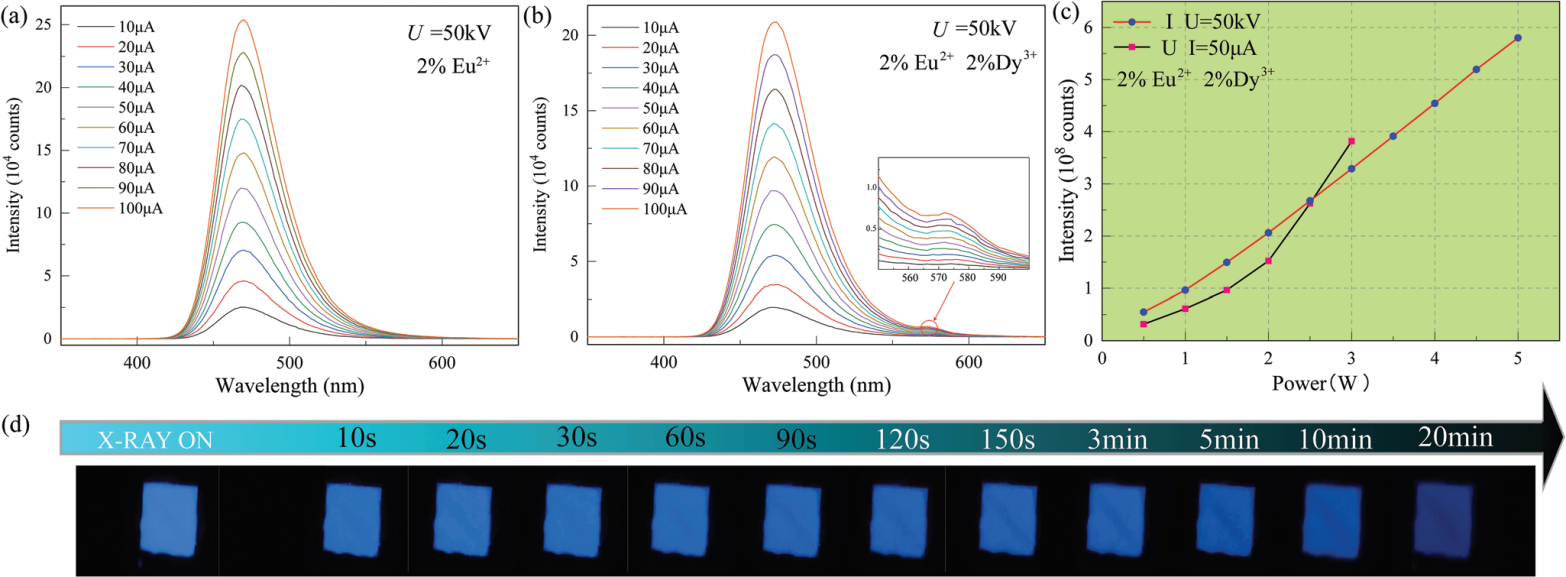
XIL spectra of SMSO. a) 2% mol Eu^2+^, *U* = 50 kV, *I* = 10–100 µA; b) 2% Eu^2+^/2% Dy^3+^(mol), *U* = 50 kV, *I* = 10–100 µA; c) At a fixed *U* = 50 kV for different currents or *I* = 50 µA for different voltages, the relationship between different power magnitudes and the integrated intensity of X‐rays; d) Afterglow images were acquired at different time intervals after irradiated by X‐ray.

In **Figure** **5**a,b, the fluorescence emission of SMSO under X‐ray excitation is characterized by a wide emission peak spanning 420–600 nm, similar to the emissions observed in PL, AG, and ML. Notably, when Dy^3+^ is doped into the SMSO sample alongside Eu^2+^, an additional small peak at 570 nm becomes apparent under X‐ray excitation. This distinctive peak corresponds to the characteristic emission associated with the Dy^3+^ ions.

The presence of this peak can be attributed to the higher energy of the X‐rays, enabling them to directly stimulate deeper traps within the material, bypassing shallower energy level traps. Figure 5c shows that a constant voltage results in a linear correlation between the current and the integrated intensity of X‐ray‐induced luminescence (XIL). This linear relationship indicates that the XIL intensity increases proportionally with the current when the voltage remains constant. However, when the current is held constant, the relationship between the voltage and integrated intensity becomes non‐linear. This non‐linear behavior can be attributed to the fact that as the voltage increases, the energy carried by the electrons increases. Consequently, an increasing number of electron–hole (e–h) pairs are generated in the excited sample, leading to a non‐linear association between the operating voltage and XIL intensity and the variation in its XIR spectrum at a fixed current, as shown in Figure S6 (Supporting Information).

These findings underscore the potential of SMSO incorporating Eu^2+^ and Dy^3+^ dopants as scintillator materials for X‐ray detection applications. The presence of a wide emission spectrum and an additional peak at 570 nm present avenues for tailoring the scintillation properties to suit specific requirements. Moreover, the observed linear correlation between the current and XIL intensity at a constant voltage indicates the adaptability of the SMSO as a flexible X‐ray detector. This adaptability was further enhanced by incorporating PDMS, enabling flexible composite materials to form.

**Figure 6:**
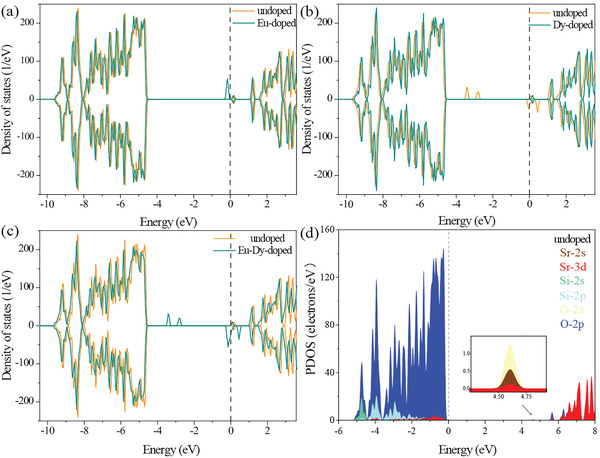
a–c) Total density of states of SMSO doped with Eu, Dy, and Eu‐Dy; d) Partial density of states of pristine SMSO.

The density of states (DOS) of undoped, Eu‐doped, Dy‐doped, and codoped SMSO is shown in **Figure** **6**, offering insights into the electronic structures and contributions of the different orbitals. The O‐2p orbitals contribute significantly more than the Sr‐2s orbitals near the Fermi level in the undoped case. Sr and dopants dominated the defect states near the Fermi level for Eu and Dy doping, demonstrating alterations in the electronic structure of SMSO. The Mg‐3s orbital primarily determined the conduction band minimum (CBM). Importantly, Eu and Dy doping directly tuned the band gap, influencing charge transport in the material. The density of state plots of undoped and doped Mg are shown in Figure S7 (Supporting Information). For Eu doping, a shallow trap emerges with a depth of 0.15 eV below the Fermi level. Conversely, the introduction of Dy creates two deep traps at 3.31 and 2.70 eV below the Fermi level, in addition to a shallow trap. Notably, the shallow defect states were enhanced by Eu and Dy codoping. These spin‐polarized defect states at the Fermi level lead to the semi‐metallic behavior of the SMSO. As shown in Figure S7 (Supporting Information), in the codoping scenario with Eu and Dy, the introduction of Eu doping gives rise to traps that transition from the upspin to the downspin direction. However, the deep traps introduced by the Dy remained unaffected. This investigation provides valuable insights into the influence of doping on the electronic structure and trap levels of SMSO. This underscores the intricate interplay between dopants and their consequent effects on the material properties.

**Figure 7:**
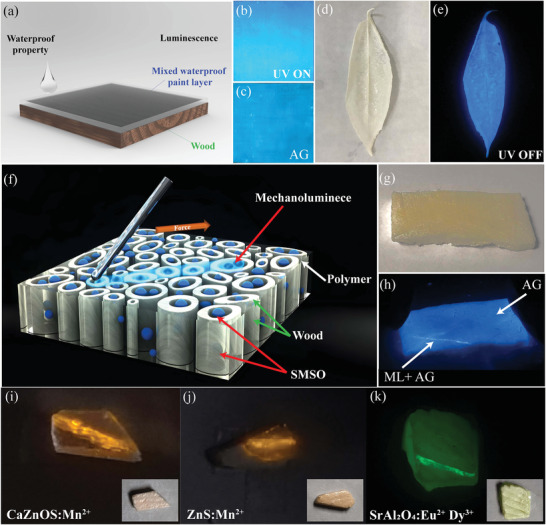
Development of SMSO optical wood. a) Mixing 2% Eu^2+^/2% Dy^3+^ powder with the paint and brushing it onto the wood panel to get both a waterproof and luminous protective layer; b) Waterproof SMSO wood is placed under UV lamp; c) Afterglow effect of the wood panel seconds after turning off the UV lamp; d) SMSO leaf under fluorescent lamp; e) Afterglow effect of SMSO leaf after turning off the lamp; f) Schematic of optical SMSO wood; g) Optical SMSO wood under fluorescent lamp; h) ML and afterglow effect of transparent SMSO wood. The multi‐functionally mechanoluminescent wood could be applied into other famous smart micro‐phosphors such as i) CaZnOS, j) ZnS, and k) SrAl_2_O_4_.

To demonstrate the potential application of SMSO as an ML material in the lighting and building decoration industry, we developed a series of wood products incorporating SMSO. These innovative products showcase the distinctive properties of SMSO, including their remarkable afterglow effect and mechanoluminescence. To enhance the practical utility and aesthetic appeal of these wood products, we successfully synthesized a new transparent paint formulation with the most pronounced afterglow intensity using SMSO powder. This specially formulated paint serves as an effective protective coating, conferring superior resistance to the erosive effects of rainfall. Consequently, the service life of wooden items, such as flooring, signs, and chairs, is significantly extended. Furthermore, owing to the exceptional environmental stability exhibited by the SMSO, the incorporated paint demonstrates an enduring and steady glow even when exposed to natural surroundings, as illustrated in **Figure** **7**a–c. By applying this paint onto wood panels, we can achieve a dual purpose: water protection and a luminescent effect. Remarkably, this effect is not limited solely to UV excitation but extends to the broad excitation peak of SMSO. Consequently, the painted surface is responsive to various light sources, including LED‐based devices, such as cell phones, light bulbs, and natural daylight. Thus, this versatile paint has considerable potential for various applications. This could serve as an effective means of waterproofing outdoor building materials, thereby expanding the utilization of wood under diverse environmental conditions. Moreover, this paint contributes to aesthetic appeal by enhancing the ornamental value during the daytime. Crucially, it fulfills a practical purpose by providing lighting guidance in dim outdoor settings. This luminescent paint acts as a guiding sign, illuminating the surroundings at night and ensuring safe pathways for individuals. In addition, we successfully developed luminous leaves by selectively eliminating lignin from fallen leaves while preserving the cellulose structure, which served as a support matrix. As shown in Figure 7d,e, these modified leaves were combined with PDMS blended with SMSO to attain a striking luminous effect. These engineered leaves are environmentally friendly, cost‐effective, and well‐adapted to the environment. Wood has garnered significant attention as an abundant resource because of its exceptional stability and durability. Consequently, researchers have pursued various methods to modify wood, catering to its versatile applications in diverse industries, particularly the construction sector. One common approach involves chemical modification achieved through delignification. This method effectively yielded nanofibrous white wood that retained its inherent wood structure and cellulose components. As a result of this chemical treatment, the mechanical, thermal, and optical properties of the wood were significantly enhanced, as shown in Figure 7f. The results of this modification process are particularly notable for the production of transparent wood. This transparent variant preserves the natural wood structure and finds utility across various construction applications and other relevant fields. Its exceptional physical properties render it a compelling material offering unique advantages for various practical applications.

The production of optical wood involves a meticulous selection process in which wood specimens displaying a transverse orientation are selected. This orientation implies that the cut surface is perpendicular to the direction of cellulose channel transmission within the wood structure. This specific orientation is advantageous for the subsequent stages of the modification process, facilitating the removal of lignin and the infusion of epoxy resin. Chemical modification techniques have been employed to selectively remove the lignin component, leaving only the cellulose framework as a structural scaffold behind. This removal process creates empty spaces within the wood structure, effectively separating the cellulose fibers. Subsequently, a vacuum‐assisted infusion technique was used to introduce a combination of epoxy resin and SMSO into the wood matrix. The outcome of these sequential processes is the generation of a novel variant of wood that exhibits unique properties such as ML and afterglow. This transformation can be observed in Figure 7g,h, showing the distinct fluorescence phenomena exhibited by the mechanoluminscent wood after modification. We performed SEM on the surface of the wood and the cross‐section (Figure S8, Supporting Information). SEM of the wood surface revealed that the epoxy resin entered the cellulose channel through one hole. The cross‐sectional image shows that the epoxy resin completely entered the channel because the SMSO particles wrapped by the epoxy resin could not be observed visually. We also performed an EDS analysis on the cross‐section to show that the SMSO successfully entered the channel. The EDS results reveal that the wood and epoxy resin components were uniformly distributed inside channel C and contained Sr, Si, Mg, and other SMSO components. This proves that SMSO successfully entered the pipe and is evenly distributed. In addition, we conducted a synthesis experiment wherein we infused the wood solely with epoxy resin, resulting in a highly transparent state, as depicted in Figure S9 (Supporting Information). The multi‐functionally wood could be applied into other famous smart mechanoluminescent phosphors such as CaZnOS, ZnS, and SrAl_2_O_4_, as shown in Figure 7i–k respectively.

## 3 Conclusion and Prospective

A comprehensive analysis of the multimode luminescence properties, including PL, LAG, XIL, and ML were conducted through mono‐ and codoping of Eu^2+^ and Dy^3+^ ions at various concentrations. These phosphors display commendable properties, including superior luminescence stability, waterproofing characteristics, and repeatability. Density‐of‐state maps obtained through first‐principles calculations were used to confirm that the improved mechanoluminescence properties of SMSO primarily originated from deep trapping. Water resistance tests demonstrated that SMSO powders exhibit stable luminescence properties in aqueous environments. We demostrate the innovative integration of the multi‐responsive SMSO into transparent wood. This multi‐functionally mechanoluminescent wood could be applied into other famous smart micro‐phosphors such as CaZnOS, ZnS, and SrAl_2_O_4_. As a new type of multifunctional smart wood, it offers promise for various applications in the fields of self‐diagnosis buildings and energy‐efficient lighting and displays.

Upon appropriate charging, the wood emits consistent and vibrant visible light, thereby serving a dual purpose as both a light source and a structural element. When subjected to stress‐induced sliding, or exposed to ultraviolet or X‐rays, the wood emits visible and intense luminescence, indicating its exceptional multi‐responsive characteristics. This unique feature makes it well‐suited for diverse applications. The development of multi‐luminescent wood is a novel and promising solution for saving the electricity consumption of environmental lighting to a certain extent. The utilization of ML wood offers significant prospects for stress detection, so the luminescence effect of this wood serves as an effective hazard warning monitor when exposed to substantial stress and radiation. In addition to its luminescent properties, this multi‐luminous wood to be improved to exhibit exceptional strength and water and fire resistance, making it highly suitable for construction and lighting applications as well as tribo‐energy harvesting and mechanical‐sensing. In the future, it will consider further improving the process to enhance the brightness of luminescence, maintain the long‐afterglow brightness caused by stress, which warrants the dynamic luminous effect under all kinds of mechanical actions such as walking, friction, sliding, and impact forces, which will considerably expand its applications in various fields. If we combine the mechanical characteristics of the wood (or bamboo) and the unique properties of mechano‐ luminescence, it would be a perfect combination of performance in many directions. The idea would open avenues for developing natural hybrids for emerging lighting and sensing applications.

## Conflict of interest

The authors declare no conflict of interest.

## Supporting information

Supporting Information

## Data Availability

The data that support the findings of this study are available from the corresponding author upon reasonable request.
