# Supplementary material for: Modulating Smart Mechanoluminescent Phosphors for Multistimuli Responsive Optical Wood
Source: Adv Sci (Weinh). 2023 Nov 8;11(1):2305066. doi: 10.1002/advs.202305066 (PMC10767394; doi:10.1002/advs.202305066)
Supplement: Supplementary file 1 — Supporting Information [file ADVS-11-2305066-s001.pdf]

ADVANCED SCIENCE
Open Access

Supporting Information

for *Adv. Sci.*, DOI 10.1002/advs.202305066

Modulating Smart Mechanoluminescent Phosphors for Multistimuli Responsive Optical Wood

Jiangcheng Luo, Biyun Ren, Xianhui Zhang, Mingju Zhu, Tianlong Liang, Zefeng Huang, Yuantian Zheng, Xu Li, Jianwei Li, Zitong Zheng, Bing Chen, Yu Fu, Dong Tu, Yu Wang, Yanmin Jia and Dengfeng Peng**

Supplementary materials

Modulating Smart Mechanoluminescent Phosphors for Multistimuli Responsive Optical Wood

Jiangcheng Luo[1], Biyun Ren[1], Xianhui Zhang[1], Mingju Zhu[1], Tianlong Liang[1], Zefeng Huang[1], Yuantian Zheng[1], Xu Li[1], Jianwei Li[1], Zitong Zheng[1], Bing Chen[1], Yu Fu[1], Dong Tu[2*], Yu Wang[3], Dengfeng Peng[1*]

J. Luo, B. Ren, X. Zhang, M. Zhu, T. Liang, Z. Huang, Y. Zhang, X. Li, J. Li, Prof. D. Peng
Key Laboratory of Optoelectronic Devices and Systems of Ministry of Education and Guangdong Province, College of Physics and Optoelectronic Engineering
Shenzhen University
Shenzhen 518060, China
E-mail: pengdengfeng@szu.edu.cn

Prof. D. Tu
Faculty of Materials Science and Chemistry, China University of Geosciences
Wuhan, 430074, China
E-mail: tudong@cug.edu.cn

Prof. Y. Wang
SZU-NUS Collaborative Innovation Center for Optoelectronic Science & Technology, International Collaborative Laboratory of 2D Materials for Optoelectronics Science and Technology of Ministry of Education, Institute of Microscale Optoelectronics
Shenzhen University,
Shenzhen, 518060, China

Experimental section

Material preparation

Samples were prepared using high-purity raw materials obtained from the following sources: $SrCO_3$ (>99%, Sinopharm Co., Ltd.), MgO (>99%, Sigma-Aldrich), SiO_2 (99.99%, Aladdin), Eu_2O_3 (99.99%, Sinopharm Co., Ltd.), and $DyCl_3 \times 6H_2O$ (99.99%, Sinopharm Co., Ltd.). The SMSO matrix was doped with Eu at various concentrations (0.5%, 0.75%, 1%, 1.5%, 2%, 3%, 4%, and 5%). The resulting homogeneous powder was collected and placed in an alumina crucible, which was then subjected to a temperature of 1400 ℃ for 5 h in a tube furnace under a protective and reducing gas mixture of N_2/H_2 (95%/5%). After annealing, the samples were naturally cooled to room temperature for subsequent characterization and testing. We used a two-step annealing process to form SMSO:Eu powders for comparison. Based on the primary annealing test, we selected a series of Eu doping molar ratios of 0.5%, 1%, 2%, 3%, 3.5%, and 4%. The original reagents and the obtained powders were annealed once and ground twice for 50 min in an agate mortar. Particularly, the first annealing process was kept under 900 ℃ in an air atmosphere for 2 h and then cooled naturally. Subsequently, the samples were remilled with alcohol for 50 min. Finally, they were sintered at 1400 ℃ for 4 h in an N_2/H_2 (95%/5%) atmosphere. The resulting samples were ground in an agate mortar, sieved through a 150 mesh sieve, and used for further characterization and application. To simplify the reaction, we still conducted primary calcination of the samples at 1100 ℃ for 2 h and then at 1400 ℃ for 6 h, both under N_2/H_2 gas atmosphere. To some extent, these calcination steps were performed to ensure the original shape of the generated powders. We used a small amount of chloride as the molten

reagent instead of boric acid to improve the reaction. This is because boric acid makes the reaction of the ceramic products relatively difficult. The selection of $DyCl_3$ was based on its ability to aid the melting process and achieve a more complete reaction, as determined by test comparisons. It is to be noted that based on the latter test, it was determined that a doping concentration of 2% Eu^{2+} was optimal. For doping with Dy^{3+}, the following concentrations were used: 0.01%, 0.1%, 0.5%, 1%, 2%, 4%, 6%, and 8% of Dy^{3+}. The preparation process for the Dy^{3+}-doped powder was identical to the secondary annealing process used for Eu^{2+} doping.

A solution with a 10% molar ratio of NaOH was prepared in a beaker to prepare ML leaves. Natural Fagaceae leaves were washed with water and soaked in an H_2O_2 solution for 30 min. Subsequently, the slides were rinsed twice with water and bleached with 30% H_2O_2 for 30 min. The leaves were rinsed with water again, followed by drying. Subsequently, a mixture of PDMS and 20 wt.% SMSO powder was prepared, and the leaves were fully submerged in the colloid. The treated leaves were removed from the colloid and placed in an 80 ℃ oven to dry. As for the SMSO wood, the chemicals used to remove the lignin content were NaOH (>95%, Sigma-Aldrich), Na_2SO_3 (>98%, Sigma-Aldrich), and hydrogen peroxide (30% solution, EMD Millipore Corporation). An epoxy resin (SpeciFix-40 resin and hardener, Struers) was used as the permeating polymer. Ethanol and deionized water were used as solvents. The lignin removal solution was prepared by dissolving NaOH (2.5 mol/L) and Na_2SO_3 (0.5 mol/L) in deionized water. The wood and lignin-removal solutions were placed in a reaction kettle and left for 6 h. The wood was then removed and rinsed twice with deionized water. This rinsing process was repeated twice to remove most chemicals. Subsequently, the wood was placed in an H_2O_2

bleaching solution and heated on a heating table at 100 ℃ until the sample turned white. The samples were then soaked in cold water, washed, and stored in ethanol. To create the SMSO wood composite, epoxy resin was mixed with a curing agent in a ratio of 2:1. The mixture was then combined with 30 wt.% SMSO powder. The white wood obtained in the previous steps was soaked in a resin mixture. Vacuum degassing was performed to remove alcohol and gas from the wood. This process lasted for 10 min, followed by vacuum release. These steps were repeated three times. Finally, the samples were placed in a 35 ℃ oven for further processing. To evaluate the ML characteristics under elevated pressure conditions, we prepared samples of SMSO by blending it with an optical epoxy resin within a plastic mold (15×25 mm). Subsequently, the mixture was cured in a drying oven. For the purpose of conducting ML measurements under high-pressure conditions.

Materials characterization

XRD patterns were recorded using a Bruker D2 phase X-ray diffraction analyzer. SEM images were obtained using a 3 Hitachi SU 8020 scanning electron microscope

Optical testing instrument

PL spectra, including long afterglow testing, were measured using an Edinburgh 28 FLS1000 spectrometer. The ML emission spectra were recorded using a homemade measurement system consisting of a linear motor, digital push-pull gauge, and QE65pro fiber optic spectrometer (Ocean Optics). In the ML test, we used a centrifuge tube to weigh out 0.3 g of the sample powder, 0.06 g of ultraviolet (UV)-curable glue and added 9 mL of anhydrous alcohol. The constituents were shaken, mixed thoroughly, and dispersed in an ultrasonic bath.

Finally, using the suspension deposition method, the sample was evenly distributed in a 3 cm × 3 cm area on an EVA-PET plastic encapsulation film (Deli No. 3817). After the alcohol was volatilized entirely, the sample was irradiated and cured using a UV lamp (LEAFTOP 9307) to obtain an ML test piece for the follow-up ML test. This membrane exhibited enhanced pressure resistance and was capable of withstanding pressures of up to 100 N and friction. This satisfied the diverse pressure testing requirements necessary for conducting the experiments. The ML pellets were initially subjected to UV light irradiation at a wavelength of 365 nm for a duration of 1 minute. After 3min, we quantified the ML intensity while applying a mechanical load using a custom-designed experimental setup. This setup comprised a universal testing machine (AGS-X10kN, Shimadzu Corp., Japan) and a photomultiplier tube (C13796, Hamamatsu Photonics, Japan). The universal testing machine was responsible for exerting the mechanical load, while the photomultiplier tube was employed to detect the resulting ML intensity. Additionally, we conducted an analysis of the ML spectrum using a fiber spectrometer (QE Pro, Ocean Optics).

Theoretical calculations

All the calculations were implemented using the VASP code [1]. The GGA-PBE functional was selected for the exchange and correlation potential [2]. Weak van der Waals interactions were considered using the DFT-D3 functional [3]. The cut-off energy for the plane wave was 400 eV. The Gamma point in the Brillouin zone was selected for integration. The total energies of the systems converge to 10–5 eV in the iteration solution of the Kohn-Sham equation. The force on each atom reduces to 0.03 eV/Å after geometry optimization. A supercell consisting of a 2 × 2 × 3 unit cell containing 288 bits was built to calculate the electronic properties of the Eu and Dy-doped SMSO. The locations of the dopants were compared for the Mg and Sr sites.

Reference
[1] Kresse G and Furthmuller J 1996 *Phys. Rev.* B 54 11169
[2] Perdew J P, Burke K and Ernzerhof M 1996 Phys. Rev. Lett. 77 3865
[3] Grimme S, Antony J, Ehrlich S, and Krieg H 2010 J. Chem. Phys. 132 154104

Table S1. Comparison of the basic parameters of the standard spectrum and SMSO obtained after XRD refinement

**Samples**	**PDF#75-1736**	**SMSO 2%Eu^{2+}**	**SMSO 2%Eu^{2+} 2%Dy^{3+}**
**Space group**	P-421m	P-421m	P-421m
**a=b (Å)**	8.0110	8.0108	8.0106
**c (Å)**	5.1630	5.1647	5.1629
**α=β=γ (°)**	90	90	90
**V (Å^3)**	331.3413	331.3011	331.3031
**Rwp**	/	10.77%	12.59%
**Rp**	/	7.9%	9.8%
**χ^2**	/	2.24	1.7

Table S2. Quantum Yield (QY) at Different Doping Concentrations.

Calcination times	Doping	Ex(nm)	Em(nm)	QY
**1**	3%Eu^{2+}	355	417~686	70.77%
**2**	0.5%Eu^{2+}	355	420~698	64.38%
**2**	1%Eu^{2+}	355	420~698	65.99%
**2**	2%Eu^{2+}	355	420~698	63.16%
**2**	3%Eu^{2+}	355	420~698	64.04%
**2**	4%Eu^{2+}	355	420~698	69.76%
**2**	2%Eu^{2+} 0.1%Dy^{3+}	355	420~715	54.48%
**2**	2%Eu^{2+} 0.5%Dy^{3+}	355	420~715	50.86%
**2**	2%Eu^{2+} 1%Dy^{3+}	355	420~715	48.30%
**2**	2%Eu^{2+} 2%Dy^{3+}	355	420~715	57.27%
**2**	2%Eu^{2+} 4%Dy^{3+}	355	420~715	59.90%

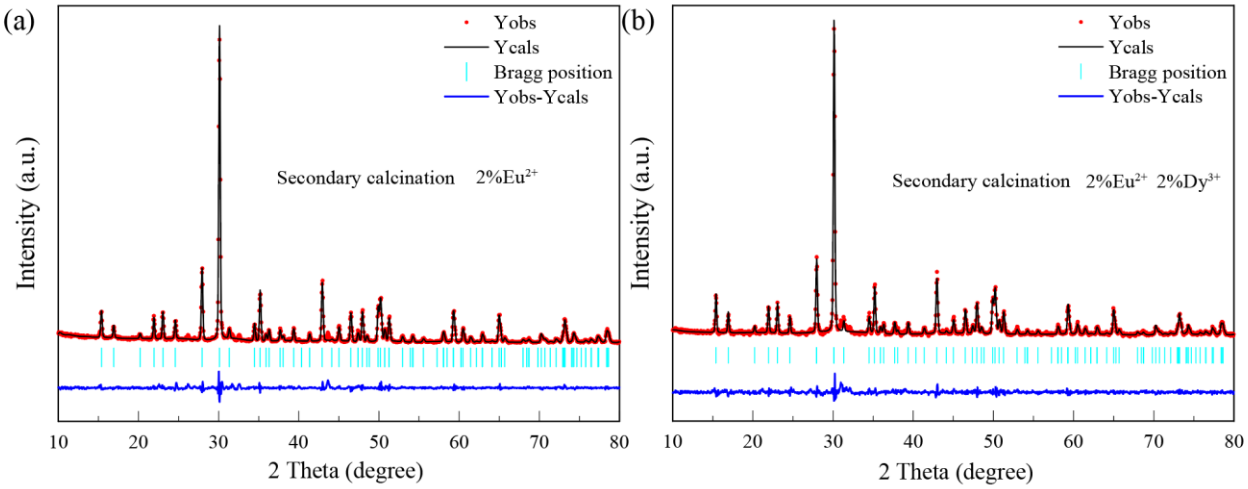

Figure S1. a) Rietveld refinement of XRD pattern of the SMSO: 2% Eu^{2+} phosphors; b) Rietveld refinement of XRD pattern of the SMSO: 2% Eu^{2+}/ 2% Dy^{3+} phosphors.

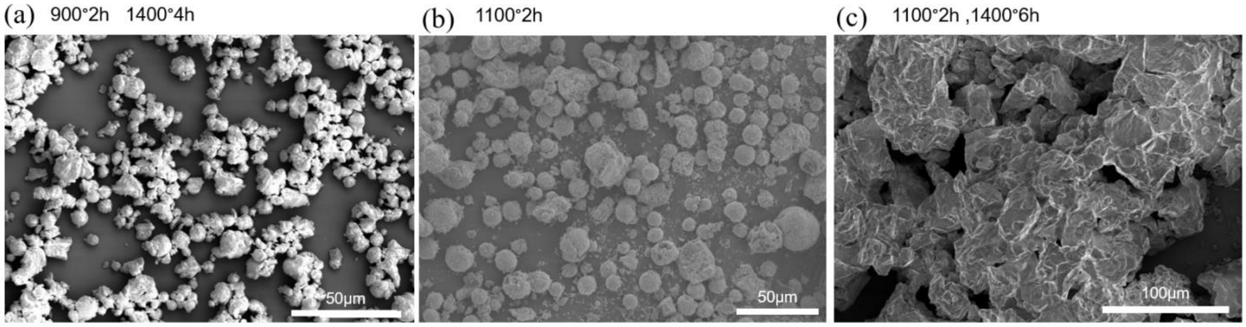

Figure S2. a) SEM of calcination at 900 °C in air for 2h followed by 1400 °C in reducing atmosphere, b) SEM of calcination at 1100 °C in reducing atmosphere for two hours c) SEM of calcination at 1100° in reducing atmosphere for 2h followed by 1400 °C in reducing atmosphere for 6h.

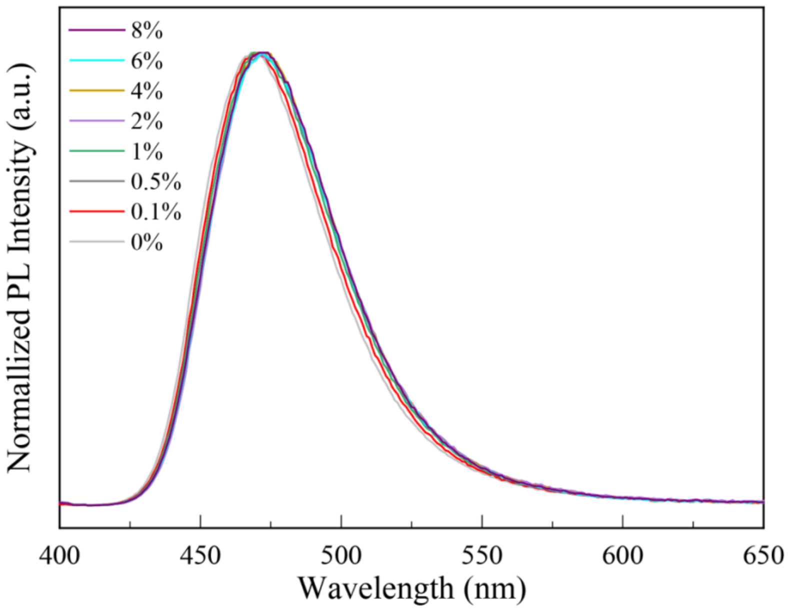

Figure S3. The normalized photoluminescence (PL) emission spectra at different Dy doping concentrations.

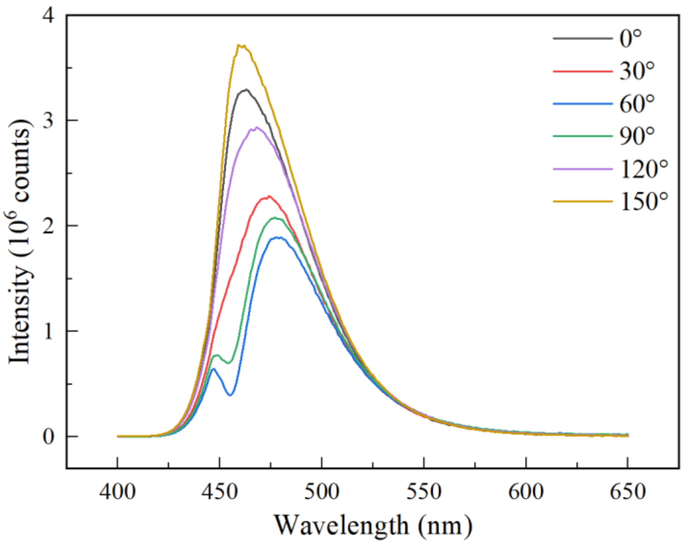

Figure S4. Plot of PL spectrum versus line polarizer angle.

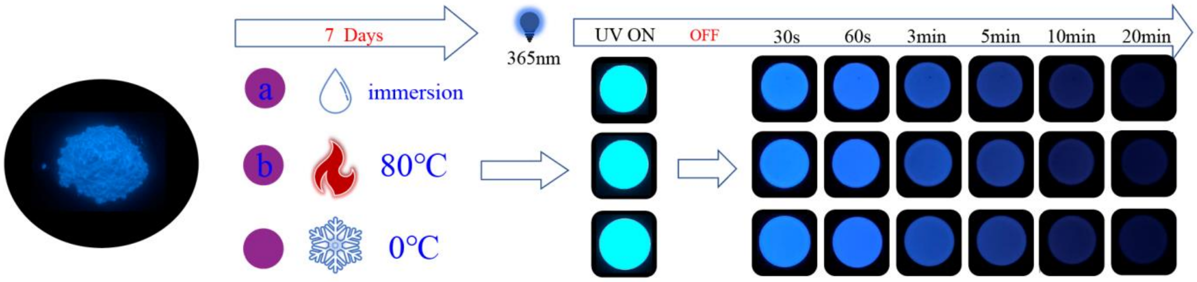

Figure S5. Physical images of samples with 2% mol Eu^{2+}/2% mol Dy^{3+}, after soaking, high temperature, and low temperature treatment for 7 days, and after 1min irradiation at 365nm.

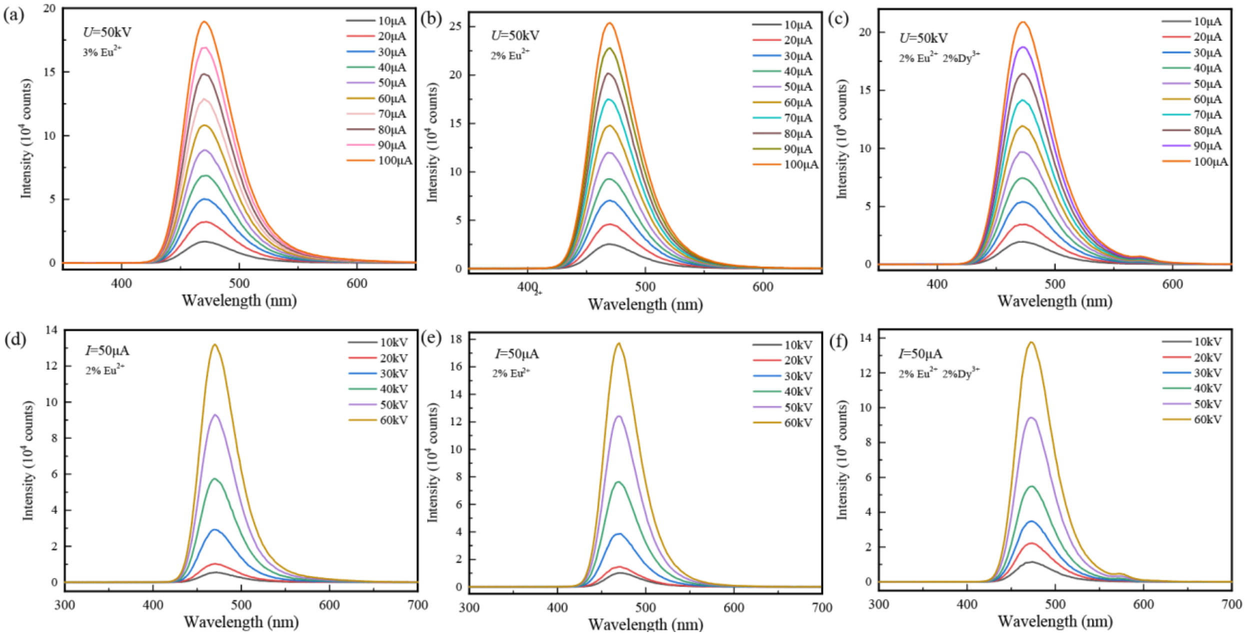

Figure S6. XIL sepectra of SMSO a) one-step calcination 3% Eu^{2+} U=50kV I=10-100μA ,b) two-step calcination 2% Eu^{2+} U=50kV I=10-100μA ,c) 2% Eu^{2+}/2% Dy^{3+} U=50kV I=10-100μA ,d) 3% Eu^{2+} I=50μA U=10-60μA , e) 2% Eu^{2+} I=50μA U=10-60μA f) 2% Eu^{2+}/2% Dy^{3+} I=50μA U=10-60μA.

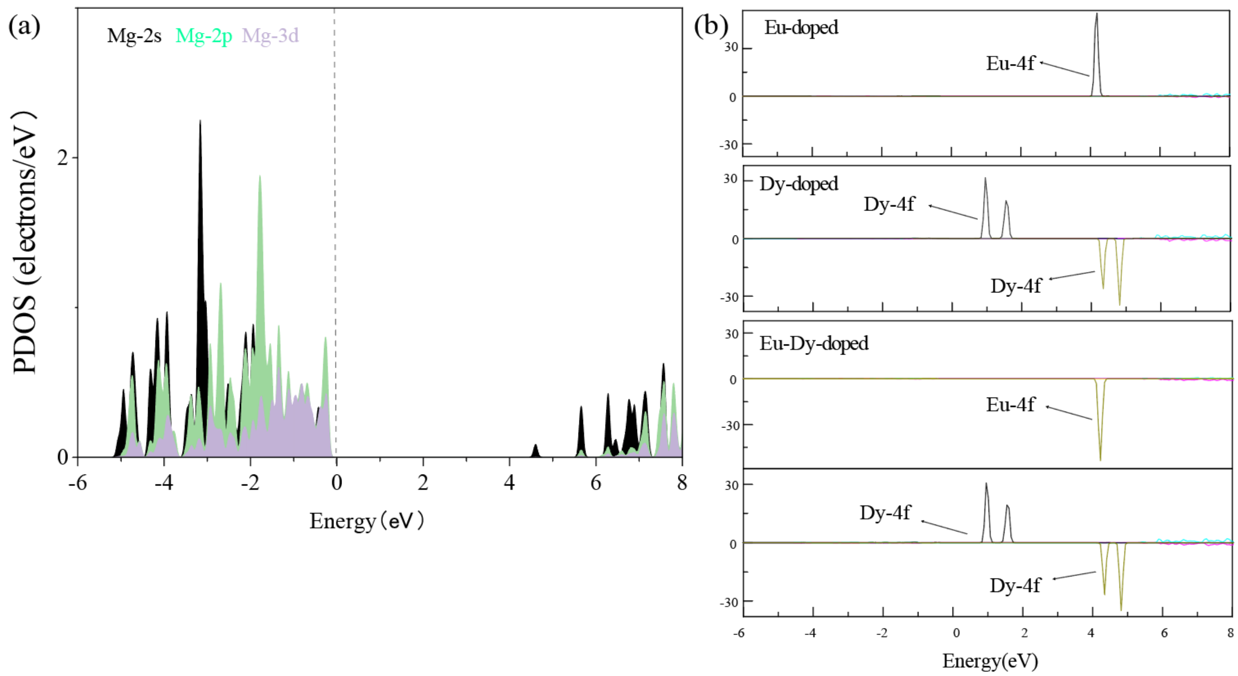

Figure S7. a) Partial density of states plots of Mg under undoped ,b) PDOS plots of doped elements under Eu-doping, Dy-doping, Eu-Dy-doping.

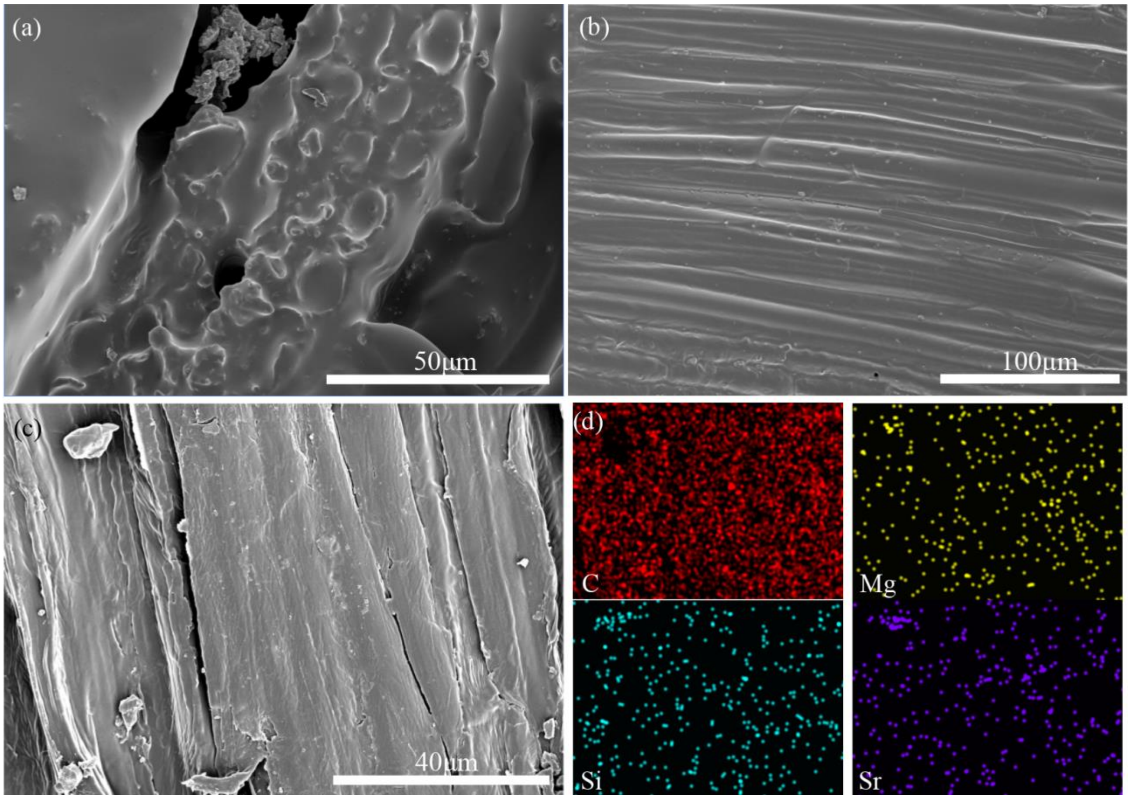

Figure S8. Typical SEM image of the Wood (a) Perpendicular to the direction of cellulose (b)and (c)Parallel to the direction of cellulose.(e) Energy-dispersive X-ray images of (c).

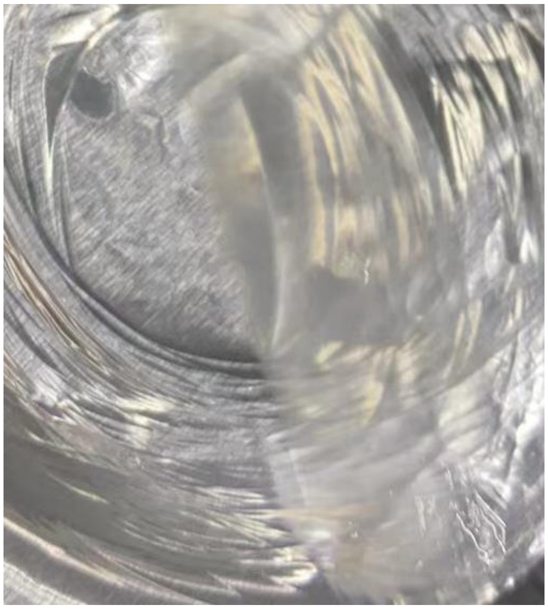

Figure S9. Uninfused SMSO synthetic transparent wood
